# Supplementary material for: Time evolution of the behaviour of Brazilian legislative Representatives using a complex network approach
Source: PLoS One. 2020 Feb 5;15(2):e0226504. doi: 10.1371/journal.pone.0226504 (PMC7001948; doi:10.1371/journal.pone.0226504)
Supplement: S1 File — Data mining and manipulation of roll-call votes sequences, Minimal Spanning Tree computation, correlation distribution fitting parameters and null model. (PDF) [file pone.0226504.s001.pdf]

Supporting information for Time evolution of the behaviour of Brazilian legislative Representatives using a complex network approach.

1 Data mining and manipulation.

The data mining consisted of processing individual files containing the results of the roll-call votes of Deputies for each session over 16 years. After identifying each elected Deputy in all sessions per year, a file containing each Deputy name and an associated array of her/his votes, was obtained for each year. Special care was taken to account for partial and total absence frequency of a Deputy in each year. The partial absence zones were filled using information about substitutes for each Deputy. If the information about the substitute of Deputy was scarce, we assume that substitute was a member of the same political party and then, if the absence zones and vote zones coincide, the substitute vote is computed. Deputies with total absences throughout the year without certified substitute were eliminated. The quantity of Deputies and bills after the data processing for each year is shown in Table S1.

Year	Deputies	Bills	Year	Deputies	Bills
2003	415	151	2011	481	101
2004	399	106	2012	466	82
2005	375	79	2013	462	161
2006	368	96	2014	455	88
2007	499	219	2015	481	300
2008	492	150	2016	400	222
2009	472	165	2017	397	236
2010	477	89	2018	391	149

Table S1: Quantity of Deputies and bills after the transfer of absence zones at the Brazilian Chamber of Deputies from 2003 to 2018. Absences were completed with the certified substitute.

2 The Minimal Spanning Tree (MST).

We use a weighted graph $G(V, E, W, f)$ for designing all networks. We employ the Prim's algorithm to obtain the MST. The Prim algorithm j^{th} step consists of finding the closest pair of vertices to set of $(j-1)^{th}$ MST sub network N_{j-1} for each only one vertex, but not the other, belongs to N_{j-1}. This way the vertex that did not belong to N_{j-1} is

now incorporated into the N_j MST subnetwork. It does not matter that another much closer pair of vertices exists, but none of the vertices are yet connected. As the algorithm interacts in a complete network, eventually, all pairs of vertices will be connected. This shows that MST connections can be the best way to connect the closest neighbours and display the clusters in the correlation, or distance, matrices. Our procedure, therefore, was to use the correlation matrix to obtain the MST and then reorder rows and columns of the correlation matrix with the MST-prim order. All MST networks were designed using the software package *Pajek* and we rearrange vertices manually to avoid much line crossing. Fig S1 shows the optimize Deputies' network from 2003 to 2018.

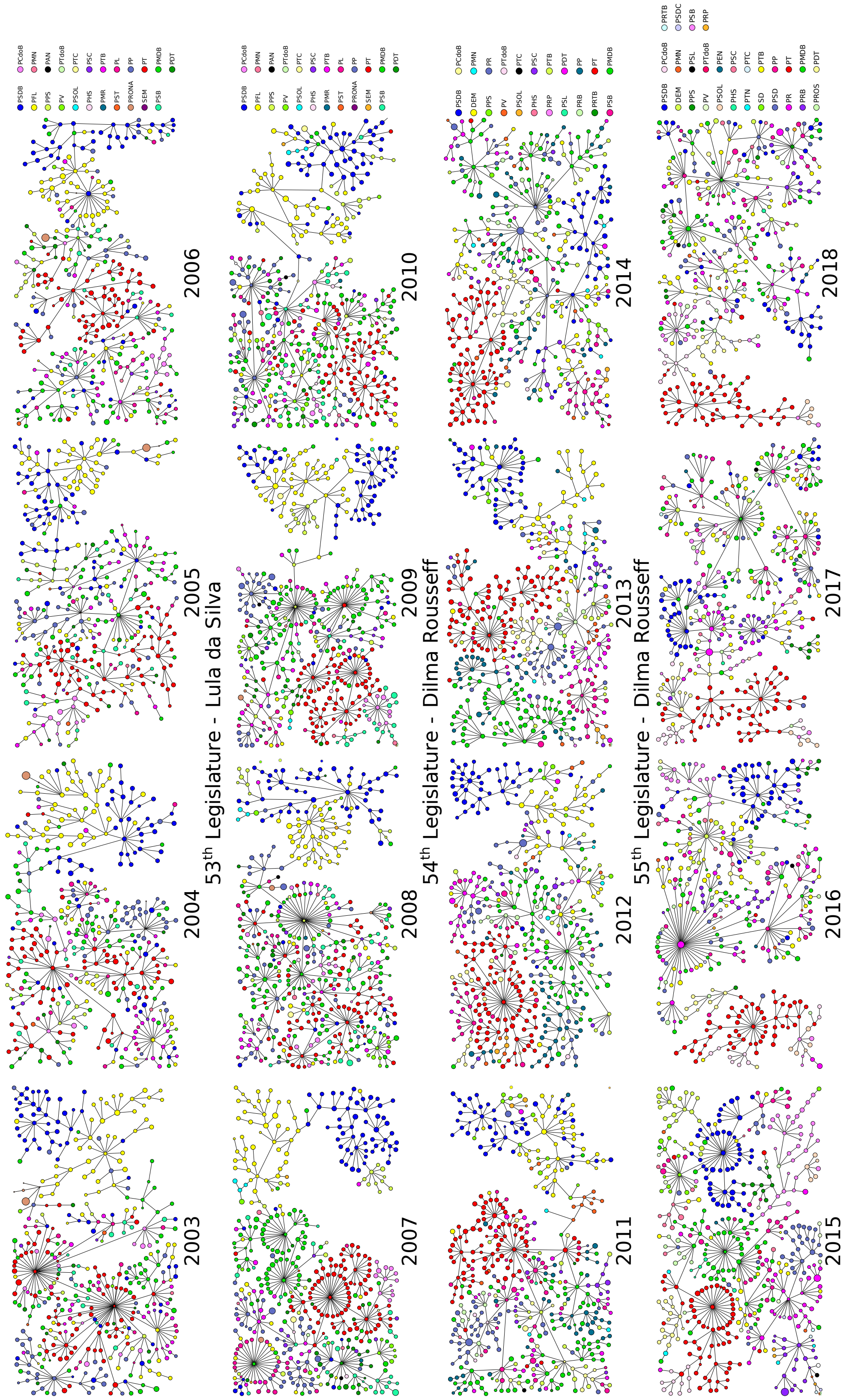

Fig S1: Representatives' networks from 2003 to 2018. Each vertex of the MST corresponds to a Deputy. Two attributes was assigned to the vertex set: the colour cluster is associated with the political party and the size of each vertex is a measure of the quantity of votes by which each Deputy was elected.

3 Correlation distribution fitting.

The fitting was made over the cumulative distribution because this distribution is independent of the choice of the bin range. The procedure was carried out by minimizing the sum of residual squared between the fitting function and the experimental data. Table S2 shows the fitted parameters of the cumulative distribution for each year.

**Year**	b	μ_1	μ_2	σ_1	σ_2	R^2
2003	0.4402	0.0816	0.6177	0.1055	0.0943	0.9984
2004	0.5491	0.2427	0.5635	0.0954	0.0830	0.9999
2005	0.4275	0.5246	0.2109	0.0810	0.0993	0.9999
2006	0.5131	0.2683	0.5374	0.0831	0.0808	0.9998
2007	0.4003	-0.0887	0.6302	0.119	0.1386	0.9992
2008	0.3951	0.0353	0.5911	0.1215	0.1463	0.9998
2009	0.4259	0.1338	0.4845	0.1225	0.1286	1
2010	0.5655	0.2596	0.4936	0.163	0.1283	0.9999
2011	0.3757	0.0029	0.6138	0.2387	0.1482	0.999
2012	0.3751	0.2086	0.4303	0.1574	0.1525	1
2013	0.8386	0.3628	0.1935	0.1407	0.1171	1
2014	0.5043	0.4430	0.2844	0.1332	0.1368	1
2015	0.9125	0.1921	0.4152	0.2346	0.0997	1
2016	0.5965	0.5418	0.6582	0.0744	0.0886	0.9998
2017	0.4164	0.1098	0.4801	0.0795	0.0892	0.9998
2018	0.4975	0.5371	0.1407	0.0863	0.0893	0.9997

Table S2: Fitted parameters for the correlation distribution for 52th, 53th, 54th and 55th Legislature of the Brazilian Chamber of Deputies.

4 Null Model

The null model was created by shuffling the roll-call vote matrices for all years. The shuffling was performed by selecting a source node, creating a list with the indexes of all connected nodes (original targets, in this case all other nodes in the network) in a random order, and replacing each edge connecting with the original targets with the ones from the random list. This process is repeated for all nodes. Fig S2 shows the MST sub-network and MST-ordered correlation matrices after the shuffling process for the 53th Legislature.

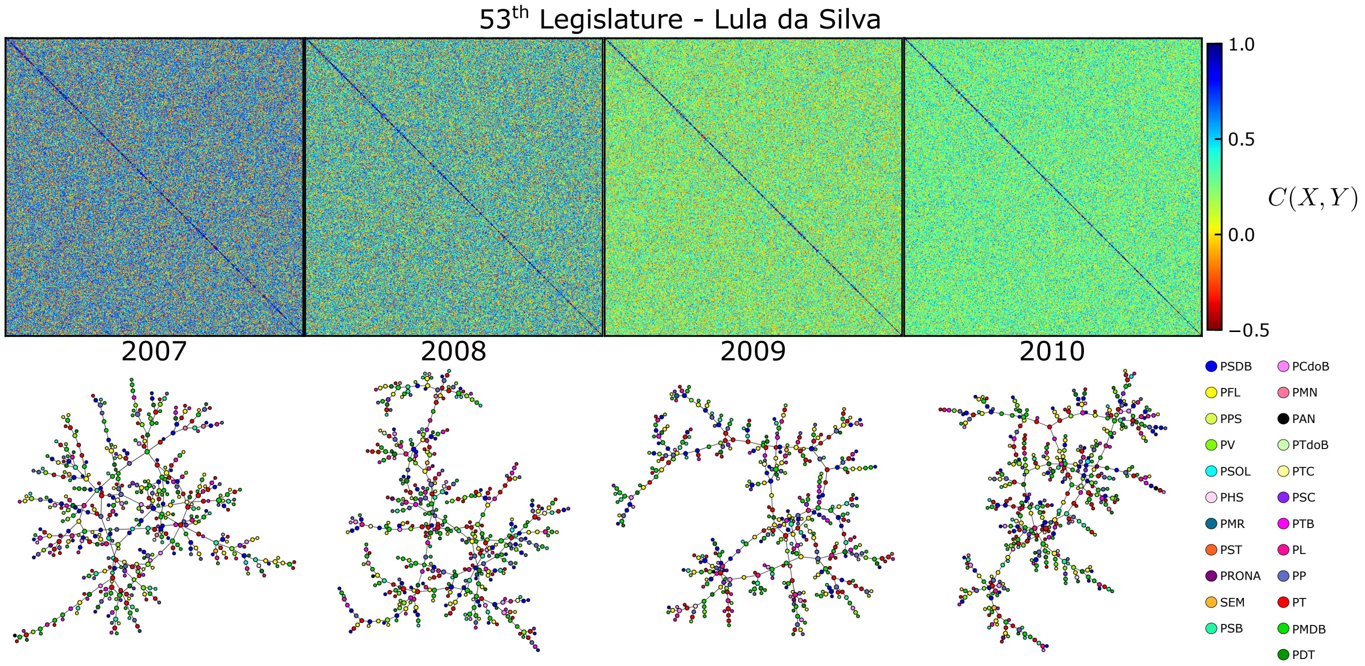

Fig S2: MST sub-network and MST-reordered correlation matrices for 53th Legislature for the Null Model of the Brazilian Chamber of Deputies.
